# Physiological and biochemical responses of soybean plants inoculated with Arbuscular mycorrhizal fungi and *Bradyrhizobium* under drought stress

**DOI:** 10.1186/s12870-021-02949-z

**Published:** 2021-04-22

**Authors:** Mohamed S. Sheteiwy, Dina Fathi Ismail Ali, You-Cai Xiong, Marian Brestic, Milan Skalicky, Yousef Alhaj Hamoud, Zaid Ulhassan, Hiba Shaghaleh, Hamada AbdElgawad, Muhammad Farooq, Anket Sharma, Ahmed M. El-Sawah

**Affiliations:** 1 Department of Agronomy, Faculty of Agriculture, Mansoura University, Mansoura, 35516 Egypt; 2 Salt-Soil Agricultural Center, Institute of Agriculture Resources and Environment, Jiangsu Academy of Agricultural Sciences (JAAS), Nanjing, 210014 China; 3 Department of Agricultural Microbiology, Faculty of Agriculture, Mansoura University, Mansoura, 35516 Egypt; 4 State Key Laboratory of Grassland Agro-ecosystems, Institute of Arid Agroecology, School of Life Sciences, Lanzhou University, Lanzhou, 730000 China; 5 Department of Botany and Plant Physiology, Faculty of Agrobiology, Food and Natural Resources, Czech University of Life Sciences Prague, Kamycka 129, 16500 Prague, Czech Republic; 6 Department of Plant Physiology, Slovak University of Agriculture, 94911 Nitra, Slovakia; 7 College of Agricultural Science and Engineering, Hohai University, Nanjing, China; 8 Institute of Crop Science and Zhejiang Key Laboratory of Crop Germplasm, Zhejiang University, Hangzhou, 310058 China; 9 College of Chemical Engineering, Nanjing Forestry University, Nanjing, 210037 China; 10 Department of Botany, Faculty of Science, University of Beni-Suef, Beni-Suef, 62511 Egypt; 11 Department of Plant Sciences, College of Agricultural and Marine Sciences, Sultan Qaboos University, 123 Al-Khoud, Oman; 12 State Key Laboratory of Silviculture, Zhejiang A&F University, Hangzhou, China

**Keywords:** AMF, Secondary metabolism, Proline metabolism, Soil enzymes, Soybean yield, Flow cytometry

## Abstract

**Background:**

The present study aims to study the effects of biofertilizers potential of Arbuscular Mycorrhizal Fungi (AMF) and *Bradyrhizobium japonicum* (*B. japonicum*) strains on yield and growth of drought stressed soybean (Giza 111) plants at early pod stage (50 days from sowing, R3) and seed development stage (90 days from sowing, R5).

**Results:**

Highest plant biomass, leaf chlorophyll content, nodulation, and grain yield were observed in the unstressed plants as compared with water stressed-plants at R3 and R5 stages. At soil rhizosphere level, AMF and *B. japonicum* treatments improved bacterial counts and the activities of the enzymes (dehydrogenase and phosphatase) under well-watered and drought stress conditions. Irrespective of the drought effects, AMF and *B. japonicum* treatments improved the growth and yield of soybean under both drought (restrained irrigation) and adequately-watered conditions as compared with untreated plants. The current study revealed that AMF and *B. japonicum* improved catalase (CAT) and peroxidase (POD) in the seeds, and a reverse trend was observed in case of malonaldehyde (MDA) and proline under drought stress. The relative expression of the *CAT* and *POD* genes was up-regulated by the application of biofertilizers treatments under drought stress condition. Interestingly a reverse trend was observed in the case of the relative expression of the genes involved in the proline metabolism such as *P5CS*, *P5CR*, *PDH,* and *P5CDH* under the same conditions. The present study suggests that biofertilizers diminished the inhibitory effect of drought stress on cell development and resulted in a shorter time for DNA accumulation and the cycle of cell division. There were notable changes in the activities of enzymes involved in the secondary metabolism and expression levels of *GmSPS1*, *GmSuSy,* and *GmC-INV* in the plants treated with biofertilizers and exposed to the drought stress at both R3 and R5 stages. These changes in the activities of secondary metabolism and their transcriptional levels caused by biofertilizers may contribute to increasing soybean tolerance to drought stress.

**Conclusions:**

The results of this study suggest that application of biofertilizers to soybean plants is a promising approach to alleviate drought stress effects on growth performance of soybean plants. The integrated application of biofertilizers may help to obtain improved resilience of the agro ecosystems to adverse impacts of climate change and help to improve soil fertility and plant growth under drought stress.

**Supplementary Information:**

The online version contains supplementary material available at 10.1186/s12870-021-02949-z.

## Background

Increasing water scarcity is a great challenge for food production worldwide especially in arid and semiarid areas [1]. Egypt is one of the most countries that are facing water deficit constrain, which are compounded recently due to the rapid increase in populations combined with a fixed share of the Nile River water (55.5 billion cubic meters per year). Therefore, developing new technologies to integrate with modern agriculture practices is of great to be used as an alternative strategy for sustainable agriculture in Egypt. Soybean (*Glycine max* L.) is an important legume crop and consider one of the most valuable oilseed crops in the world, being contains about 18–22% cholesterol-free oil with 85% unsaturated fatty acids and 40–42% protein [2, 3]. Recent studies reported that adequate water supply are needed for soybean production to achieve high yield [4, 5]. However, plants can tolerate water stress only up to a certain limit (threshold level) and beyond that limit, there is a severe decline in yield [6]. In soybean case, it was reported that at 40% field capacity, plants experience severe drought stress [7]. It exists due to low moisture in the soil at a certain time, therefore the available water for plants is limited [8]. Previous studies have shown that water deficit reduced soybean yield by 40% as compared to the well-watered conditions [9–11]. Hence, all plant physiological processes such as cell turgidity, photosynthetic processes, growth of the cell, tissue, and organs are influenced by drought stress [12, 13].

As such, drought stress can significantly decrease the contents of the chlorophyll a, b, and total chlorophyll in soybean leaves [14, 15], and can accumulate higher proline content in the plants [16, 17]. Additionally, the antioxidant enzymes such as POD, and CAT in soybeans were highly activated under abiotic stresses [18–20], specifically under water stress [21], to adapt, control, and to scavenge the free radicals induced by drought stress and accompanied higher accumulation of MDA [14]. The previous study has shown that the biomass production of soybean was decreased under drought conditions [22], and that the negative impacts of the water deficit varied depending on the growth stage in which the soybean was exposed to the drought stress.

In this context**,** exposing soybean plants to a moderate drought for a short time during the vegetative stage increases the acclimation of the plants to the drought in later developmental stages [23, 24]. Similar reports have shown that soybean yield was not adversely affected when the plants were exposed to moderate soil water deficit only for a short period of time during the vegetative stage [25]. In another study, soybean plants that experienced drought before flowering produced higher seed yields than plants that were stressed after flowering, as the plants that were exposed to the drought at the early growth stage had established a bigger root system before flowering as an acclimation response [26]. Accordingly, during flowering and pod filling stages, soybean plants are very sensitive to drought stress [4]. However, water deficit during the pod-enlargement and seed-filling stages has a significant negative effect on the final yield and the yield components [27]. Thus, it is an urgent issue to develop practical strategies for reducing the adverse impacts of the water deficit on the production of soybean.

Plant growth-promoting microorganisms such as *B. japonicum* and AMF are one of the most promising strategies used to enhance plant growth by improving nutrient availability to the plant through biological nitrogen fixation and phosphate solubilization processes in the soil and rhizosphere [28, 29]. These microorganisms can also alleviate the stress effects through the modulation of 1-aminocyclopropane-1-carboxylate deaminase expression, and inducing phytohormonal signals [30]. Duc et al. [31] indicated that AMF inoculation, particularly with *Septoglomus constrictum* alleviated the adverse impacts of drought and heat stress on tomato plant. Moreover, AMF can enhance plant tolerance to various environmental stresses by improving the mineral nutrient and water acquisition and thus enhance crop yield [32, 33], and can also affect the water balance of both amply watered and drought-stressed host plants [34]. The AMF can interfere with the stomatal conductance and thus improve the drought stress tolerance [35], as well as decrease the oxidative damage by stimulated higher enzymatic and non-enzymatic antioxidant activities [36]. Furthermore, AMF have been reported to regulate patterns of expression of aquaporin genes [37], and altered proline content in leaf tissue [38] It is well known that leguminous plants can establish specific symbiosis relationship with rhizobia such as *Rhizobium*, *Bradyrhizobium*, *Sinorhizobium*, *Azorhizobium*, *Mesorhizobium*, and *Allorhizobium* [39]. These rhizobia play important roles in agriculture, due to their capability for biological nitrogen fixation and consequently reduce the need for chemical nitrogen fertilizers [40]. However, several factors related to the host plant, bacterial species, and edaphic soil variables, especially drought, can restrict the contribution of nitrogen fixation to plant growth performance [41]. A recent study showed that inoculation of the soil with *Rhizobium* strains increased nodulation, nitrogen assimilation, and legume yield [42]. Moreover, *Bradyrhizobium japonicum* has effectively improved soybean growth, nodulation, nitrogen fixation, the acquisition of nitrogen, phosphorus, and potassium (NPK), and seed yield [43].

The hypothesis of the present study was that *B. japonicum* and AMF could increase the availability of mineral nutrients to soybean plants and alleviate the water deficit effects on soybean growth. Therefore, a complete analysis of the main physiological, biochemical and molecular mechanisms involved the response of soybean plants to drought with respect to their production traits, photosynthesis, and metabolites to elucidate the underlying mechanisms regulating adaptation of soybean plant to drought stress. Moreover, the antioxidant enzymes and proline metabolism and their corresponding genes were also investigated to elucidate further the potential capability of AMF and rhizobia to improve soybean growth under the drought stress condition. Thus, this study was to understand, evaluate, and maximize the use of microbial inoculation to provide a theoretical and practical basis for its application as an alternative technology for fertilization for high-yield, drought-resistant soybean cultivation under water deficient regions.

## Methods

### Experimental site

**Fig. 1:**
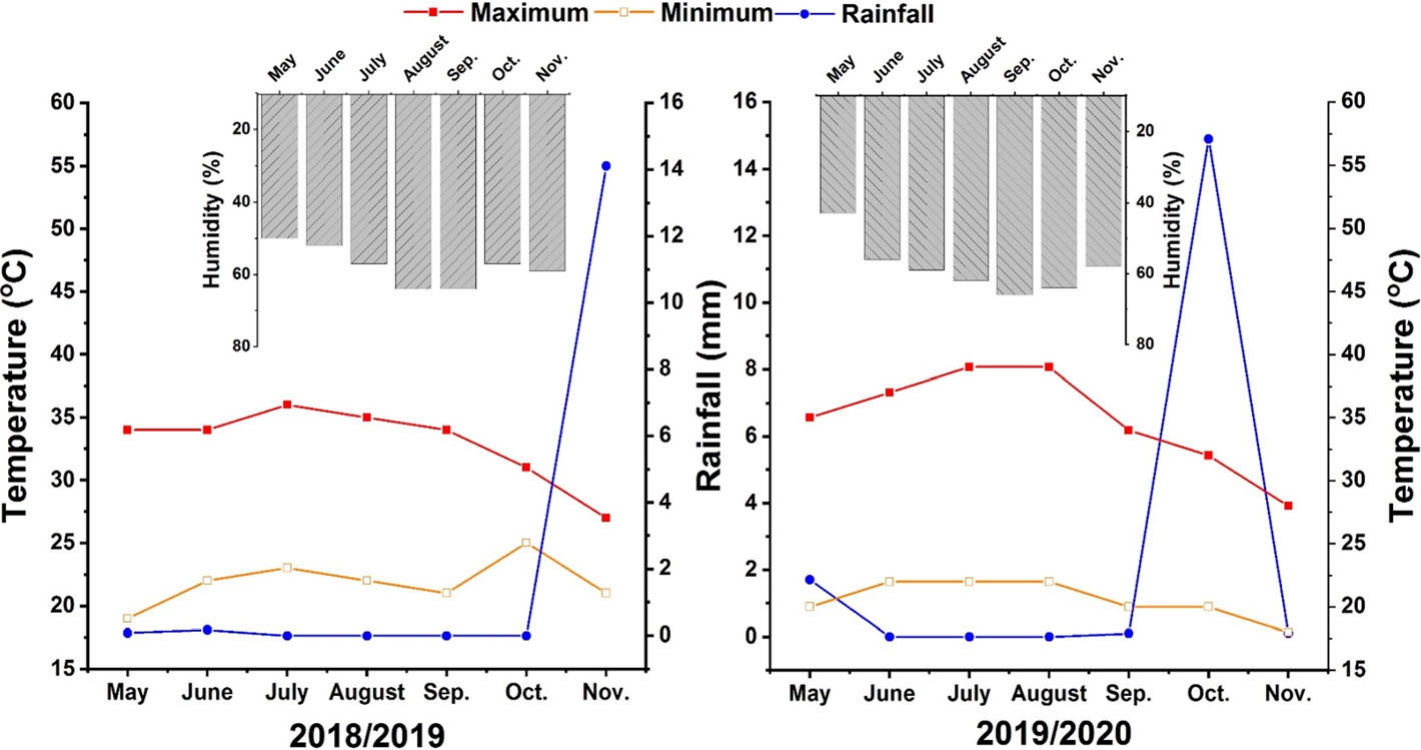
Meteorological data of temperature (°C) and relative humidity (%) during 2018 and 2019 growing seasons

Field experiments were conducted for 2 years during 2018 and 2019 at the Agronomy Farm of Mansoura University, Egypt (27.00^°^N, 30.00°E) from May to November. Figure 1 presents the meteorological data in the study region during both seasons. The soil texture was clay loam, Table 1 shows some physicochemical and biological properties of experimental soil.

**Table 1:** Physicochemical and biological properties of soil used in 2018 and 2019 growing seasons

Property	2018/2019	2019/2020
pH	7.89	8.84
OM %	1.55	1.76
EC	1.73	1.53
**Cations (meq L**^**− 1**^**)**		
Ca++	7.89	17.11
Mg++	4.06	7.89
Na+	7.20	12.52
K+	0.45	0.72
**Anions (meq L**^**−1**^**)**		
CO_3_^−^	0.00	0.00
HCO_3_^−^	3.07	2.36
Cl^−^	11.02	16.95
SO_4_^−^	5.52	18.93
**Available nutrients (PPM)**		
N	35.0	154.0
P	6.19	7.33
K	177.45	246.48
**Bacterial count**		
TBC	6.181	4.795
PDBC	6.259	4.869

pH (1:2.5); *OM* Organic Matter; *EC* (electrical conductivity dsm^−1^); *TBC* total bacterial count log (cfu g^− 1^ dry soil) and *PDBC* phosphate dissolving bacterial count log (cfu g^− 1^ dry soil)

### Agronomic practices

The seedbed was prepared by disc plough and leveled ridging. The plot size was 3 m × 3.5 m having four ridges of 2.5 m in length. Seeds of soybean cv. Giza 111 were procured from Sakha Agricultural Research Station, Kafr El-Sheikh, Egypt. The seeds were sterilized using sodium hypochlorite then, washed with sterilized water after this, the seeds were soaked in the microbial inoculants before planting. For mycorrhizal inoculation, 5 g of trapped soil and 0.5 g of infected roots of Sudan grass were inoculated to each hill, the inoculum was placed on the depth of 5 cm below the soil surface before sowing. Thereafter, the seeds were sown (3 seeds per hill) manually on the shoulder of the 70 cm spaced ridges in 25 cm apart hills.

After 3 weeks, the plants were thinned to maintain two plants per hill. The fertilizers were applied at 180, 361, and 120 kg ha^− 1^ nitrogen (N), phosphorus (P), and potassium (K) using urea (46% N), calcium superphosphate (15.5% P_2_O_5_), and potassium sulfate (48% K_2_O), respectively as sources.

The treatments were arranged in a strip-plot design with four replicates, in which the different treatments of fertilization (without fertilization, 100% NPK, 50% NPK, *B. japonicum* + 50% NPK, Mycorrhiza + 50% NPK, *B. japonicum* + Mycorrhiza + 50% NPK) were serving as the vertical plots. While the irrigation treatments (withholding irrigation at R3 and withholding irrigation at R5) were serving as horizontal plots.

Irrigation was performed according to the optimized recommendations for soybean production in Egypt (550 mm/total growing period) and based on the local farmers’ irrigation practice for the soybean cropping system in the Mansoura region of Egypt. The irrigation was managed as the following: Plots were firstly irrigated with 10% of the total water requirements after 15 days from sowing. The following irrigation events were applied at 15 day intervals with 25% (vegetative growth), 25% (flowering R1 to early pod R3) and 35% (pod development to pod fill R4-R6) of the total water requirements during the stages of the growth, while the soil was naturally dried during the maturation (5%). To induce the drought stress, the irrigation was withheld for 2 weeks at the early pods’ stage (50 days from sowing, R3), and seed development (90 days from sowing, R5), in both seasons. Irrigation water was pumped from the pond nearby and induced through pipes to the plots, and the amount of freshwater was measured by a water meter. Soil pH was measured in 1:5 soil and water extract by using a calibrated pH meter. The weeds were controlled by hand and stomp (BASF) 500 (4 L/hectare) was also used as an herbicide to control the weeds. All the above agronomic practices were performed uniformly for all the treatments.

### Bradyrhizobium inoculum preparation

*B. japonicum* USDA-110 was obtained from the Laboratory of Bacteriology, Sakha Agricultural Research Station, Kafr El-Sheikh, Egypt. Then, the strain was tested for indole acetic acid (IAA) production in Yeast extract mannitol (YEM) medium [44], supplemented with 0.1% L-tryptophan according to the method of Ahmad et al. [45] and for phosphate solubilization in Pikovaskya liquid medium [46], supplemented with tricalcium phosphate [47]. For inoculum preparation, *B. japonicum* USDA-110 was grown on YEM medium for 5 days at 30 °C until the culture density reached (1.3 × 10^9^ cfu/mL). Soybean seeds were inoculated with *B. japonicum* culture according to the study of Gao et al. [29]. Briefly, the seeds were soaked in microbial inoculants containing arabic gum (16%) as an adhesive agent for 30 min, left to dry in the air, and then the seeds were transplanted. With the second irrigation, an additional culture (10 mL) was also added to each plant. The non-bacterial treatments received equal amounts of autoclaved inoculum to provide the same nutrients.

### Mycorrhizal inoculum preparation

AMF spores of *Glomus clarum*, *Glomus mosseae,* and *Gigaspora margarita* were obtained from Botany Department, Faculty of Science, Mansoura University, Egypt and AMF were grown for 6 months on *Sorghum sudanenses* Pers. roots as a host plant to propagate. Five grams of trapped soil containing approximately 50 spores g^− 1^ soil, and 0.5 g of infected sudan grass roots (70% colonization index) were used to inoculate mycorrhizal-treated according to the study of Asrar et al. [48].

### Staining, estimation of mycorrhizal root colonization and microbial count determination

After 120 days from planting, fresh roots of soybean plants were stained with 0.05% trypan blue [49]. The colonization levels were estimated by the method of Trouvelot et al. [50], using Mycocalc software (Wuhan, Hubei, China). The soil was separated from the roots by vigorous shaking, and then the soil was passed through a 2-mm mesh to give the bulk fraction. The remaining fine roots (< 2 mm) and soil were gently shaken in a plastic container for 1 min to separate the soil aggregates (0.5–5 mm) from the roots yielding the rhizosphere fraction [51]. In the soybean plant rhizosphere after 120 days from planting, the total bacterial count and phosphate-dissolving bacteria were counted on nutrient agar medium (oxoid, UK) and Pikovskaya medium [46] according to the method of Gao et al. [29].

### Morpho-physiological, nodulation, and grain yield

Randomly, four plants from each block were chosen on 15 September and used to measure root length, plant dry weight, number of branches/plant, and chlorophyll content. The chlorophyll content was determined on the midpoint of the youngest fully-expanded leaf using SPAD-502 (Minolta Co. Ltd., Osaka, Japan) according to our previous study [29]. While the number of nodules on the root of each plant was counted and their mean was expressed as the number of nodules per plant. At the end of the mature stage (1st of November), the plants were harvested and the grain yield per hectar was determined.

### Enzymatic extraction and biochemical analyses

The activity of dehydrogenase and acid phosphatase were measured in the rhizosphere according to Zhang et al. [52] The antioxidant enzymes such as CAT and POD were measured in the fresh seeds according to the method of Sheteiwy et al. [21] Briefly, seeds samples (0.5 g each) were homogenized in 8 mL of 50 mM potassium phosphate buffer (pH 7.8) under ice-cold conditions. Then, the homogenate was centrifuged at 10000×g for 20 min at 4 °C and the supernatant was used for POD and CAT determination according to the method of Salah et al. [53]. The activities of sucrose synthase (SuSy), sucrose phosphate synthase (SPS), and acid invertase (AI) were measured according to the method of Jiang et al. [54]. Proline was determined according to the method of Sheteiwy et al. [21]. Lipid peroxidation was measured as far as MDA content according to the method of Zhou and Leul [55].

### Flow cytometry analysis and transcription levels analysis

Nuclear isolation was performed from the seeds according to the method of Hu et al. [56]. The transcription level of the antioxidant, proline and secondary metabolism was analyzed in the fresh seeds according to our previous study [13]. Briefly, frozen seed (100 mg each) was grinded thoroughly in liquid nitrogen using a pestle and mortar. Thereafter, the total RNA was isolated from the seeds and the concentration of the RNA was determined by NanoDrop 2000/2000c (Thermo Scientific, USA). The RNA purity was also checked by the spectrophotometer with means of the 260/280 nm ratio before quantitative real time PCR. Quantitative real-time RT-PCR was performed using SYBR premix EX Taq (Takara, Japan). The PCR program used in this study are the same as used recently by Sheteiwy et al. [13]. The sequences (5′-3′) of forward (F) and reverse (R) primer of all genes are presented in supplementary Table S1.

### Statistical analysis

Differences among treatments were evaluated with two-way ANOVA considering droughts stress and biofertilizers as fixed factors. The present results are the means of three replicates ± standard deviation (SD). The data were analyzed by analysis of variance using the IBM-SPSS statistical package, (IBM-SPSS, 19, USA). Mean values were compared by applying Duncan’s multiple range test at the 0.05 level of significance between the levels of the studied factors. Asterisks indicate significant differences: *P < 0.05, **P < 0.001, ***P < 0.0001 among the studied factors.

## Results

### Plant growth promotion traits of *B. japonicum* USDA-110

**Fig. 2:**
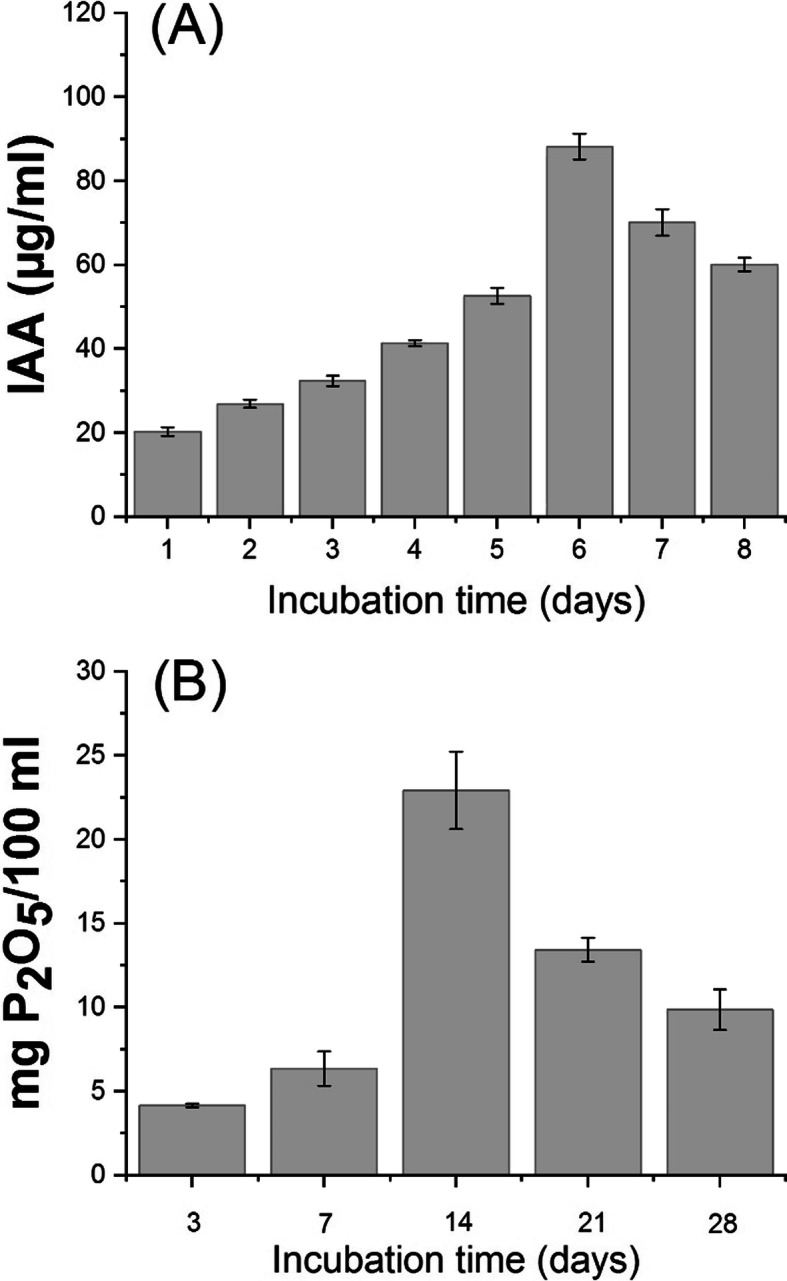
Plant growth-promoting traits of *Bradyrhizobium japonicum* USDA-110, **a**; Indole acetic acid (IAA) production (μg/ml culture) and (**b**); phosphate solubilization (mg P_2_O_5_/100 ml culture) after different time of incubation at 30 °C

The result presented in Fig. 2 showed the ability of bacterial strain (*B. japonicum* USDA-110) to produce IAA and effectively solubilize phosphate. From the first day of incubation, IAA was detected, gradually increased and reached its maximum level on the 6th day (88.20 μg/ml culture), then the IAA production was gradually decreased (Fig. 2a). As such, the maximum soluble phosphorus release was 22.92 mg P/100 mL culture after 14 days of incubation and then decreased with the advance of the incubation period (Fig. 2b).

### Effects of biofertilizers on plant biomass and chlorophyll content under drought stress

Mean data regarding the morpho-physiological traits as affected by the biofertilizers application under water stress are presented in Table 2. Soybean seedlings grown under normal irrigation have the highest root length and plant dry weight as compared with plants grown under withholding water at R3 and R5 stages in both growing seasons. The lowest values of root length, and dry weight were observed in the plants exposed to withholding irrigation at R3 stage as compared with withholding irrigation at R5 stage and the complete irrigation in both growing seasons (Table 2). Irrespective of the drought stress effects, AMF and *Bradyrhizobium* treatments improved the root length and dry weight of plants as compared with untreated plants. The lowest values of root length and dry weight were observed in the plants without biofertilizers treatment and exposed to water deficit at R3 in both growing seasons. Root length was improved with the application of *Bradyrhizobium* by 35.7, 12.1, and 29.5%; and with the application of AMF by 42.0, 20.7, and 36.8% and with their combination by 38.9, 16.46, 33.0% in both growing seasons as compared with control, 100% NPK and 50% NPK, respectively. The plant dry weight was improved with the application of *Bradyrhizobium* by 59.3, 13.5 and 34.8%; and with the application of AMF by 63.2, 21.8 and 41.0% and with their combination by 61.7, 18.7, 38.7% in both growing seasons as compared with control, 100% NPK and 50% NPK, respectively (Table 2). Both withholding irrigation at R3 and R5 resulted in a decrease of chlorophyll content as compared with the well-irrigated plants in both growing seasons. Chlorophyll content was improved with the application of *Bradyrhizobium* by 54.3, 27.1 and 35.4%; and with the application of AMF by 56.3, 33.5 and 41.5% and with their combination by 54.7, 31.1, 39.0% in both growing seasons as compared with control, 100% NPK and 50% NPK, respectively (Table 2).

**Table 2:** Influence of biofertilizers alone or in combination on plant biomass and leaf chlorophyll contents of soybean under drought stress

Treatments		2018/2019			2019/2020		
		Root length (cm)	DW/Plant (g)	Chl. content (mg L^− 1^)	Root length (cm)	DW/Plant (g)	Chl. content (mg L^− 1^)
CK	Without NPK	22.8i-l	26.5 h	19.1f	21.8hi	23.9i	17.5d
	100% NPK	30.3d-f	54.7e	26.5 cd	30.4 cd	58.8e	24.6bc
	50% NPK	25.0 g-j	42.0f	21.8ef	25.1e-h	42.4gh	21.3c
	*B. japonicum* + 50% NPK	39.3 ab	58.7c-e	36.3ab	34.1bc	66.3bc	36.4a
	Mycorrhiza+ 50% NPK	42.3a	67.8a	37.3a	45.2a	72.5a	37.8a
	Mixture + 50% NPK	37.6b	65.5ab	35.3ab	35.7b	70.5ab	37.1a
D1	Without NPK	14.7 l	23.9 h	14.9 g	18.2i	20.6i	15.8d
	100% NPK	20.7 k	42.1f	21.8ef	26.8d-h	49.2f	21.7c
	50% NPK	16.0 l	31.7 g	20.1f	18.4i	37.3 h	21.6c
	*B*. *japonicum* + 50% NPK	22.3i-k	53.1e	34.7ab	29.4c-f	57.9e	34.40a
	Mycorrhiza+ 50% NPK	25.7 g-i	57.3de	34.9ab	27.6d-g	59.4de	36.80a
	Mixture + 50% NPK	27.3f-h	53.9e	35.9ab	26.4d-h	59.3de	35.23a
D2	Without NPK	21.3jk	25.7 h	13.7 g	22.7 g-i	23.0i	14.20d
	100% NPK	27.9e-g	53.0e	24.8de	30.1c-e	47.2 fg	25.33bc
	50% NPK	23.8 h-k	37.4f	22.2ef	24.9f-h	39.2 h	21.20c
	*B*. *japonicum* + 50% NPK	31.4de	55.5de	29.16c	32.7bc	61.4c-e	27.73b
	Mycorrhiza+ 50% NPK	33.4 cd	63.6a-c	35.1ab	35.5b	69.8ab	35.93a
	Mixture + 50% NPK	35.7 cd	60.70b-d	32.7b	36.3b	65.3b-d	34.10a
**Fertilization**		***	***	***	***	***	***
**Drought**		***	***	***	***	***	**
**Fertilization × Drought**		**	**ns**	**	**	**ns**	*

Means sharing the same letters, for a parameter during a year, do not differ significantly at *α =* 0.05 after Student–Newman–Keul test; ns, not significant; and *, **, ***, denote significant differences at *P ≤ 0.05*, *0.01*, and *0.001*, respectively, among the studied factors. Mixture: (*B. japonicum* + Mycorrhiza); *DW* (Dry weight); *Chl* (Chlorophyll); *CK* (Control); *D1* [irrigation withholding at early pod stage (50 days from sowing, R3)]; *D2* [irrigation withholding at seed development stage (90 days from sowing, R3)]

Results showed that the application of *Bradyrhizobium*, AMF, and their combination improved the number of branches per plant of both drought-stressed plants and well-irrigated plants in both growing seasons (Table 3). Both withholding irrigation at R3 and R5 resulted in a decrease of the number of branches per plant as compared with the well-irrigated plants in both growing seasons. The number of branches per plants was improved with the application of *Bradyrhizobium* by 52.1, 15.3, and 35.8%; and with the application of AMF by 46.6, 5.7, and 28.5% and with their combination by 48.7, 9.3, 31.3% in both growing seasons as compared with control, 100% NPK and 50% NPK, respectively. These results suggested that biofertilizers diminished the harmful effects of drought stress by improving soybean seedlings growth through nodulation and nitrogen fixation ability of plants under stress conditions as compared with untreated plants (Table 3).

**Table 3:** Influence of biofertilizers alone or in combination on branching, nodulation and grain yield of soybean under drought stress

Treatments		2018/2019			2019/2020		
		Branches/Plant	Nodules/Plant	Grains yield (t/ha)	Branches/Plant	Nodules/Plant	Grains yield (t/ha)
CK	Without NPK	2.6ef	3.0i	1.19f	3.3ef	4.6i	1.10 h
	100% NPK	4.6a-d	24.0f	2.32bc	6.6a-c	26.3e	2.50c
	50% NPK	3.6c-f	13.3 g	1.64e	5.0b-e	15.6gh	1.68f
	*B*. *japonicum* + 50% NPK	5.6ab	59.6a	2.49b	8.6a	65.0a	2.83b
	Mycorrhiza+ 50% NPK	6.3a	33.0e	2.87a	6.0bc	35.6d	3.07a
	Mixture + 50% NPK	5.6ab	51.0b	2.50b	7.0ab	55.3b	2.63c
D1	Without NPK	2.3f	0.66i	0.98 g	2.6f	1.3i	0.96hi
	100% NPK	3.6c-f	14.0 g	2.10d	6.3bc	18.3 fg	2.22de
	50% NPK	3.0d-f	8.3 h	1.35f	4.3c-f	11.3 h	1.41 g
	*B*. *japonicum* + 50% NPK	4.3b-e	38.3d	2.20 cd	5.6b-d	44.0c	2.29d
	Mycorrhiza+ 50% NPK	3.6c-f	25.6f	2.51b	5.0b-e	27.0e	2.50c
	Mixture + 50% NPK	4.0b-f	31.6e	2.19 cd	6.0bc	33.0d	2.29d
D2	Without NPK	3.0d-f	1.3i	0.94 g	3.0ef	2.65i	0.89i
	100% NPK	4.0b-f	23.3f	2.06d	4.6b-f	21.6f	2.08e
	50% NPK	3.0d-f	11.6gh	1.37f	3.6d-f	14.3 gh	1.29 g
	*B*. *japonicum* + 50% NPK	5.0a-c	46.3c	2.14 cd	6.0bc	51.0b	2.17de
	Mycorrhiza+ 50% NPK	4.6a-d	30.6e	2.25fcd	5.6b-d	32.6d	2.28d
	Mixture + 50% NPK	4.6a-d	40.6d	2.13 cd	5.6b-d	44.3c	2.19de
**Fertilization**		***	***	***	***	***	***
**Drought**		***	***	***	**	***	***
**Fertilization × Drought**		**ns**	***	**ns**	**ns**	***	**

Means sharing the same letters, for a parameter during a year, do not differ significantly at *α =* 0.05 after Student–Newman–Keul test; ns, not significant; and *, **, ***, denote significant differences at *P ≤ 0.05*, *0.01*, and *0.001*, respectively, among the studied factors. Mixture: (*B. japonicum* + Mycorrhiza); *CK* (Control); *D1* [irrigation withholding at early pod stage (50 days from sowing, R3)]; *D2* [irrigation withholding at seed development stage (90 days from sowing, R3)]

### Effects of biofertilizers on nodulation and grain yield under drought stress

Mean data regarding nodules number and grain yield are presented in Table 3. Results revealed that inoculation with *Bradyrhizobium* and AMF and their combination has significantly increased nodules number as compared with non-inoculated plants (Table 3). Additionally, there was a decrease in nodulation under drought stress, which was reflected by the reduction in nodules number when the plant was exposed to drought stress at R3 and R5 stages, and this reduction was accentuated in plants those exposed to drought stress at R3 stage (Table 3). Similarly, grain yield also was negatively affected by withholding irrigation at both R3 and R5 stages in both growing seasons as compared with the complete irrigation. It could be stated that grain yield was improved with the application of *Bradyrhizobium* by 57.0, 5.9, and 25.9%; and with the application of AMF by 60.8, 14.3, and 32.5% and with their combination by 56.4, 4.7, 25.0% in both growing seasons as compared with control, 100% NPK and 50% NPK, respectively (Table 3).

### Effects of biofertilizers on soil enzymes under drought stress

**Fig. 3:**
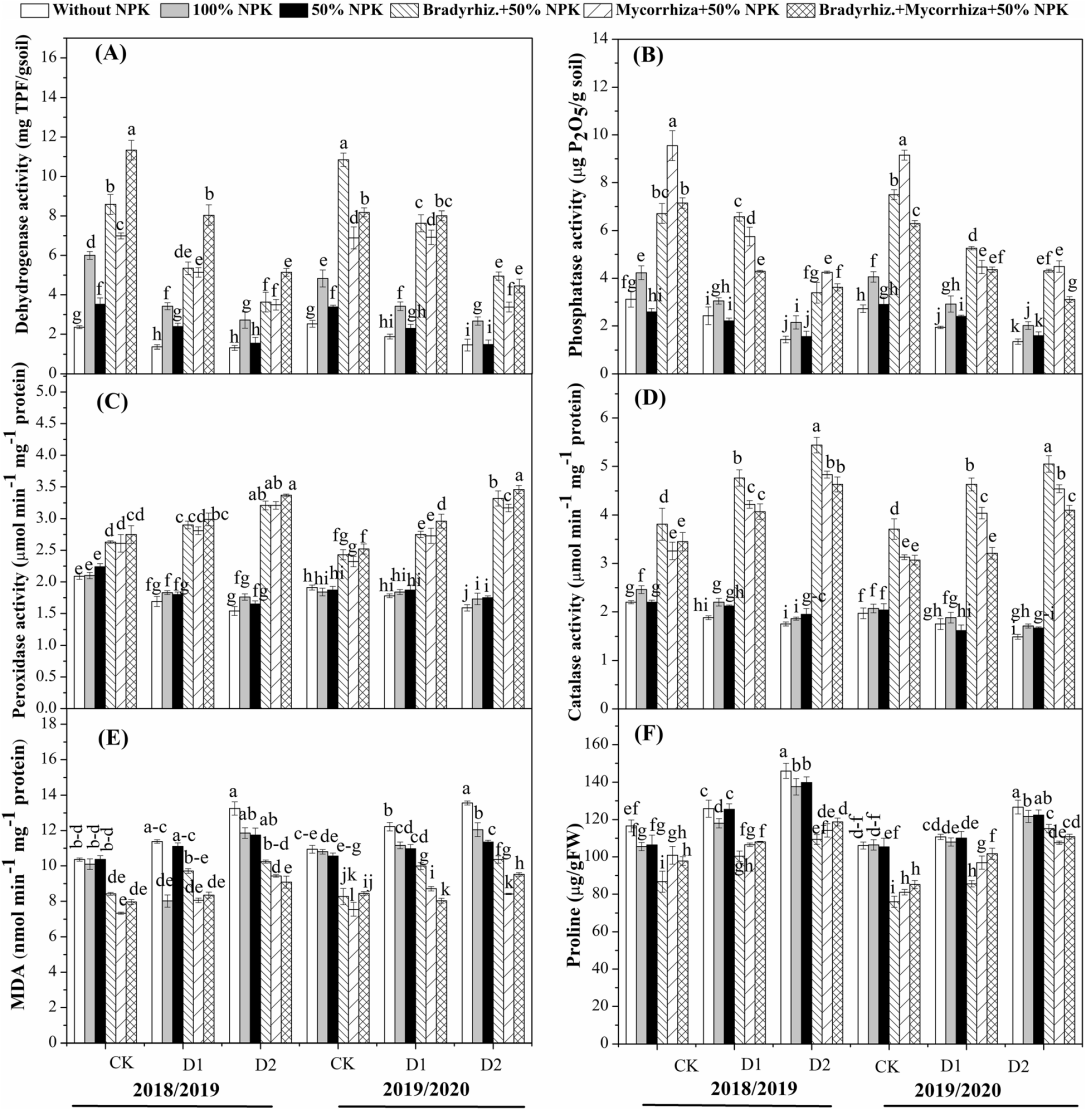
Effects of biofertilizers on the activities of dehydrogenase (**a**); phosphatase (**b**); peroxidase (**c**); catalase (**d**); and the contents of MDA (**e**) and proline (**f**) of soybean under drought stress. Means sharing the same letters, for a parameter during a year, do not differ significantly at *α =* 0.05 after Student–Newman–Keul test. Ck (complete irrigation); D1 (withholding irrigation at R3 stage) and D2 (withholding irrigation at R5 stage)

Results showed that dehydrogenase and phosphatase activities in the rhizosphere soil were decreased by withholding irrigation at R3 and R5 stages as compared with the complete irrigation in both growing seasons (Fig. 3 a and b). However, inoculation with biofertilizers has improved dehydrogenase and phosphatase activities in rhizosphere soil as compared with control plants in both growing seasons. The highest dehydrogenase activity was observed with the combination treatment in both growing seasons, while the highest values of phosphatase activity were observed with combination treatment, except for withholding irrigation at R3 stage, which was higher in the *Bradyrhizobium* -treated plants in both growing seasons (Fig. 3). Dehydrogenase activity was improved with the application of *Bradyrhizobium* by 73.4, 43.7 and 64.4%; and with the application of AMF by 66.4, 29.7 and 55.5% and with their combination by 76.1, 49.3, 67.9% in both growing seasons as compared with control, 100% NPK and 50% NPK, respectively. Phosphatase activity was improved with the application of *Bradyrhizobium* by 57.0, 5.9, and 25.9%; and with the application of AMF by 61.5, 45.3 and 60.8% and with their combination by 65.5, 51.0, 64.9% in both growing seasons as compared with control, 100% NPK and 50% NPK, respectively (Fig. 3). These findings proves that inoculation with such microorganisms resulted in an increase in microbial quantity and enzyme activity in the rhizosphere soil with the complete irrigation as well as, with drought stress.

### Effects of biofertilizers on antioxidant enzymes, proline, secondary metabolism, and MDA under drought stress

Results showed that the application of *Bradyrhizobium* and AMF and their combination resulted in the highest POD and CAT activities in the seeds upon plants were exposed to withholding irrigation at R3 and R5 stages in both growing seasons (Fig. 3). A reverse trend was observed in the case of proline and MDA in both seasons. It could be summarized that POD activity was improved with the application of *Bradyrhizobium* by 38.6, 35.5 and 35.1%; and with the application of AMF by 37.1, 33.9, and 33.5% and with their combination by 41.3, 38.3, 38.0% in both growing seasons as compared with control, 100% NPK and 50% NPK, respectively (Fig. 3c). While, CAT activity was improved with the application of *Bradyrhizobium* by 58.8, 54.5, and 56.8%; and with the application of AMF by 45.0, 49.2, and 51.7% and with their combination by 50.9, 45.8, 48.5% in both growing seasons as compared with control, 100% NPK and 50% NPK, respectively (Fig. 3d). On the contrary, MDA activity was decreased by the application of *Bradyrhizobium* by 20.5, 10.9, and 13.8%; and by the application of AMF by 31.0, 22.7, and 25.1% and with their combination by 35.8, 28.14, 30.4% in both growing seasons as compared with control, 100% NPK and 50% NPK, respectively (Fig. 3e). Similarly, proline activity was also decreased by the application of *Bradyrhizobium* by 21.9, 17.8, and 19.3%; and by the application of AMF by 17.1, 12.9, and 14.5% and with their combination by 15.0, 15.0, 12.3% in both growing seasons as compared with control, 100% NPK and 50% NPK, respectively (Fig. 3f). Thus, the current study suggested that the application of biofertilizers may have the potential to increase the antioxidant system to reduce the oxidative damage induced by the lipid peroxidation under drought stress conditions.

**Fig. 4:**
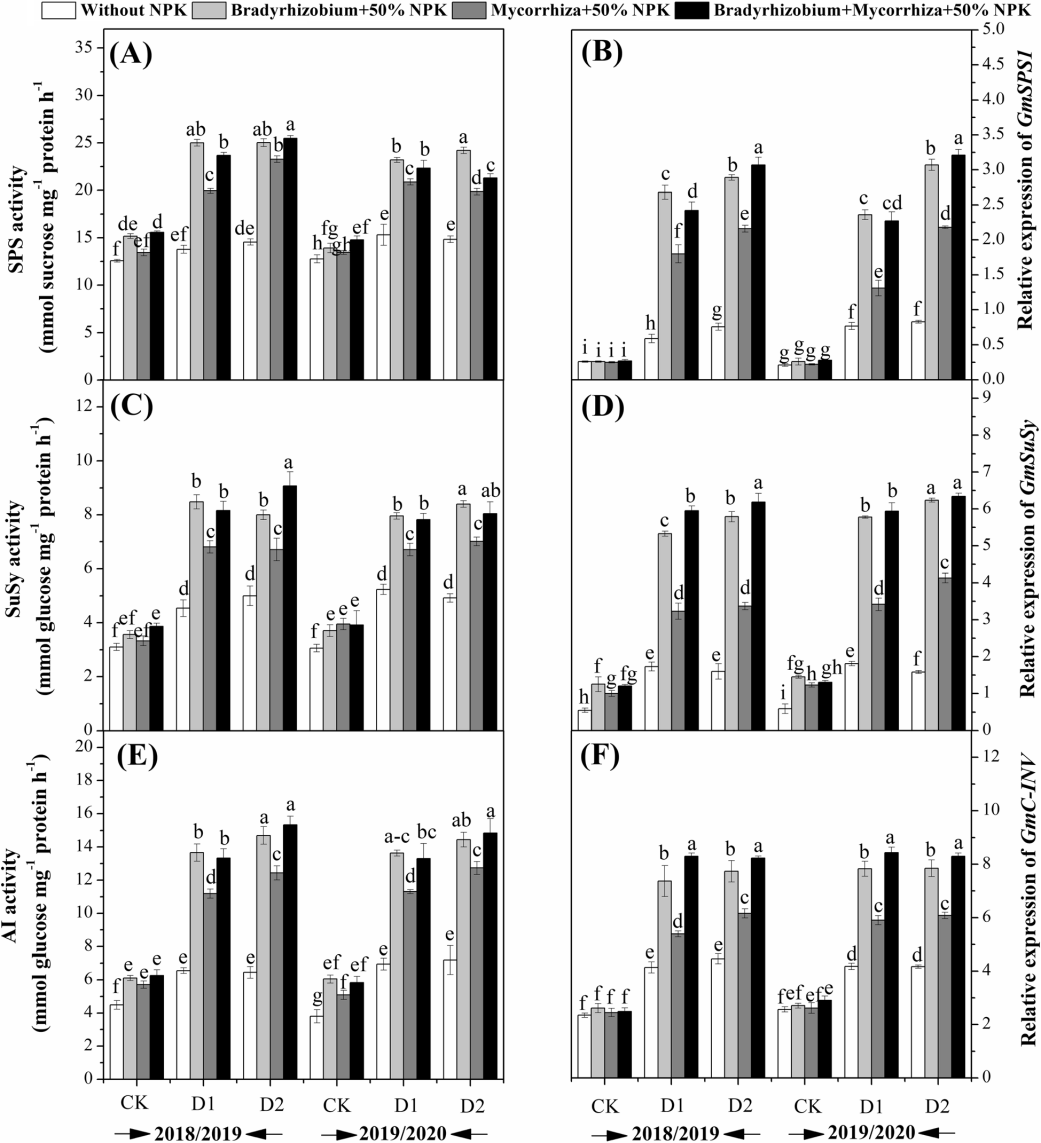
Effects of biofertilizers on the activities of SPS (**a**), SuSy (**c**), AI (**e**); relative expression level of *GmSPS1*(**b**); *GmSuSy* (**d**) and *GmC-INV* (**f**) of soybean under drought stress condition. Means sharing the same letters, for a parameter during a year, do not differ significantly at *α =* 0.05 after Student–Newman–Keul test*.* Ck (complete irrigation); D1 (withholding irrigation at R3 stage) and D2 (withholding irrigation at R5 stage)

The activities of SPS, SuSy, and AI were improved by inoculation with *Bradyrhizobium* and mycorrhiza and their combination (Figs. 4) and this was pronounced in the plants those exposed to the drought stress at R3 and R5 as compared to unstressed plants. The activities of SPS (Fig. 4a) and SuSy (Fig. 4c) were significantly improved in the plants those exposed to drought stress at R5 and treated with the combination of biofertilizers during 2018, while it was significantly improved in the plants those treated with *Bradyrhizobium* during 2019 growing season (Fig. 4a). While the activity of AI was higher in the plants those exposed to the drought stress at R5 and treated with *Bradyrhizobium* alone and the combination treatment without significant differences between them (Fig. 4e).

### Effects of biofertilizers on transcription levels of secondary metabolism, antioxidant enzymes and proline under drought stress

**Fig. 5:**
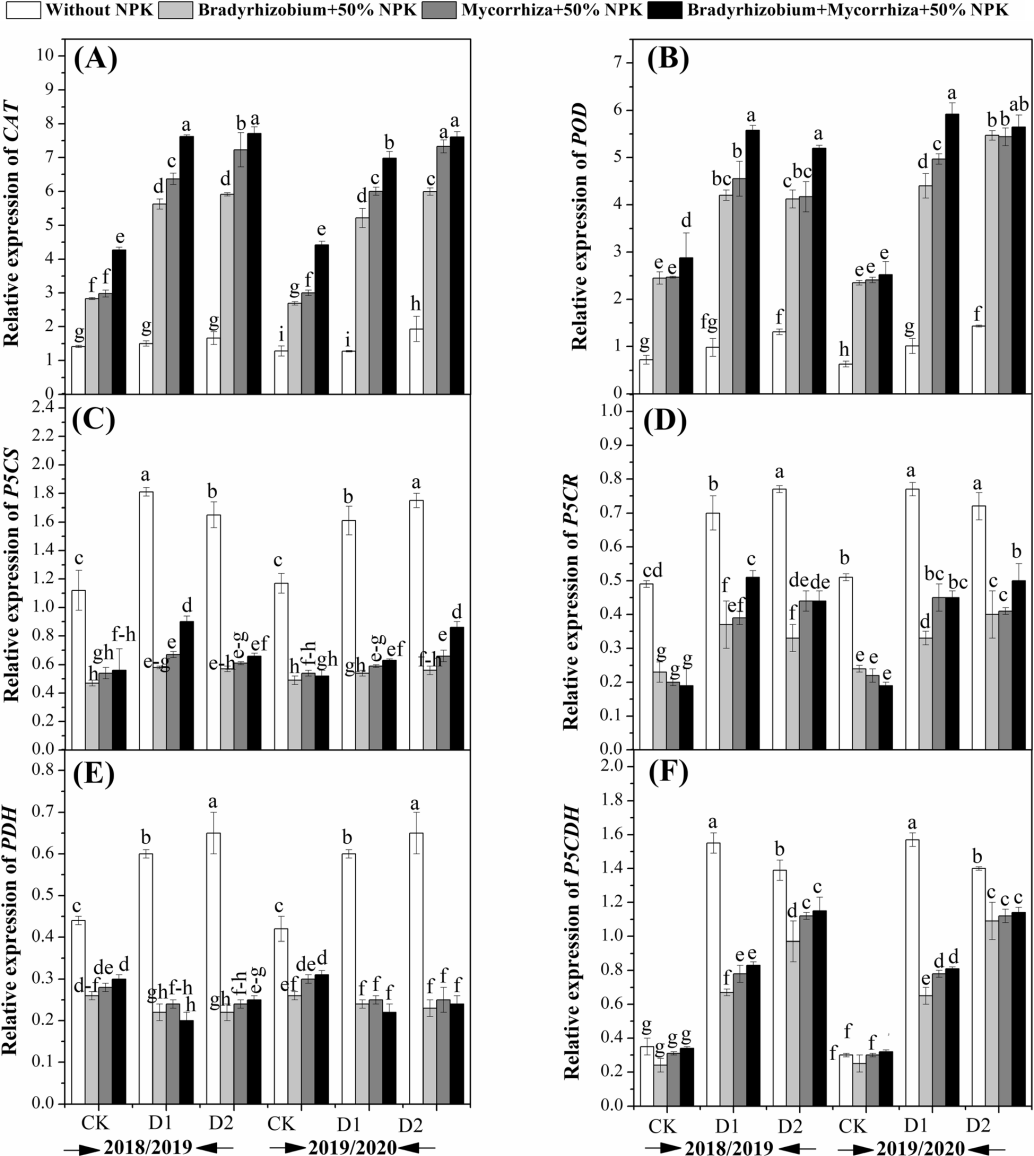
Effects of biofertilizers on the relative expression level of *CAT* (**a**); *POD* (**b**); *P5CS* (**c**); *P5CR* (**d**); *PDH* (**e**) and *P5CDH* (**f**) of soybean under drought stress condition. Means sharing the same letters, for a parameter during a year, do not differ significantly at *α =* 0.05 after Student–Newman–Keul test. Ck (complete irrigation); D1 (withholding irrigation at R3 stage) and D2 (withholding irrigation at R5 stage)

The relative expression of *GmSPS1* was up-regulated by the biofertilizers treatments under drought stress in both growing seasons (Fig. 4b). *GmSPS1* was only significantly up-regulated in the plants treated with the combination treatments and exposed to drought at R5 stage. Similarly, the *GmSuSy* expression was significantly up-regulated in the plants exposed to drought stress at R3 and R5 stages and treated with *Bradyrhizobium* and the combination treatment (Fig. 4 D). While the expression level of *GmC-INV* was significantly improved in the plants those exposed to drought stress at R3 and R5 and treated with the combination treatment in both growing seasons (Fig. 4 F). Mean data regarding the relative expression of the genes involved in the antioxidant enzymes activities and proline metabolism as affected by the application of biofertilizers and the drought stress condition are shown in Fig. 5. Results showed that *CAT* and *POD* expression levels in the seeds was up-regulated by the application of biofertilizers and drought stress effects as compared with the controlled plants in both growing seasons (Figs. 5a and b). Higher expression of *CAT* was observed at the R5 stage in the plants those treated with the combination of AMF and *Bradyrhizobium* (Fig. 5a), while the *POD* expression was found to be higher at R3 stage of the plants those treated with the AMF and *Bradyrhizobium* in both growing seasons (Fig. 5b) as compared to their respective controls. It could be concluded that the application of *Bradyrhizobium*, AMF, and their combination improved the expression level of *CAT* by 76.6, 72.6, and 67.6%, respectively, and *POD* by 78.1, 74.7, and 73.6% relative to their controls in both growing seasons. In contrast, the genes involved in the proline metabolism such as *P5CS*, *P5CR*, *PDH,* and *P5CDH* were down-regulated by the application of the biofertilizers treatments (Fig. 5c-f). It could be concluded that the application of *Bradyrhizobium*, AMF, and their combination reduced the expression level of *P5CS* by 64.9, 55.6 and 54.9%, *P5CR* by 0.53, 46.9 and 42.4%, *PDH* by 58.9, 53.5 and 55.3%, and *P5CDH* by 41.2, 33.0 and 30.2%, respectively as compared to their respective controls in both growing seasons.

### Effects of biofertilizers on the bacterial count and mycorrhizal colonization under drought stress

The effects of chemical and biofertilizers on bacterial counts isolated from the soybean rhizosphere with and without drought stress are presented in Table 4. Regardless of the effect of drought, the total bacterial counts and phosphate-solubilizing bacteria isolated from the rhizosphere of the plants treated with biofertilizers were higher than those of chemical fertilizer-treated plants (Table 4). However, drying soil and re-irrigation leads to an increase in bacterial counts during both growing seasons. The highest number of bacterial counts (total counts and P-solubilizers) was observed in the rhizosphere of plants those treated with biofertilizers and exposed to withholding irrigation R3 stage, followed by withholding irrigation at R5 stage as compared with plants without drought stress.

**Table 4:** Bacterial counts (Log (cfu g^-1^ dry soil)) in root rhizosphere and mycorrhizal colonization levels (%) in roots of soybean treated with biofertilizers alone or in combination under drought stress

Treatments		2018/2019					2019/2020				
		Bacterial counts		Mycorrhizal colonization			Bacterial counts		Mycorrhizal colonization		
		Total	P-dissolvers	F	M	A	Total	P-dissolvers	F	M	A
Ck	Without NPK	7.740 g	5.680j	–	–	–	7.781j	5.725i	–	–	–
	100% NPK	8.007e	5.994e-g	–	–	–	7.995gh	6.023ef	–	–	–
	50% NPK	7.870f	5.870hi	–	–	–	7.902i	5.930 g	–	–	–
	*B. japonicum* + 50% NPK	8.099de	6.085c-e	–	–	–	8.089ef	6.119c	–	–	–
	Mycorrhiza + 50% NPK	8.163 cd	6.091 cd	85.0b	52.3a	41.2a	8.145de	6.111 cd	83.0b	50.2a	40.2a
	Mixture + 50% NPK	8.175 cd	6.092 cd	90.0a	48.2b	38.1b	8.132ef	6.109 cd	90.0a	48.8b	39.7a
D1	Without NPK	7.861f	5.825i	–	–	–	8.002gh	5.839 h	–	–	–
	100% NPK	8.240bc	6.040d-f	–	–	–	8.220 cd	6.047de	–	–	–
	50% NPK	8.090de	5.946gh	–	–	–	8.156de	5.974 fg	–	–	–
	*B*. *japonicum* + 50% NPK	8.479a	6.296a	–	–	–	8.418a	6.221b	–	–	–
	Mycorrhiza + 50% NPK	8.472a	6.320a	75.3d	30.4e	24.8d	8.494a	6.340a	75.6e	31.3d	20.0c
	Mixture + 50% NPK	8.457a	6.289a	73.6e	29.6f	20.0f	8.474a	6.309a	72.6f	28.6e	18.5d
D2	Without NPK	7.869f	5.725j	–	–	–	7.824j	5.850 h	–	–	–
	100% NPK	8.033e	5.979 fg	–	–	–	8.053 fg	6.026ef	–	–	–
	50% NPK	7.905f	5.937gh	–	–	–	7.940hi	5.959 fg	–	–	–
	*B*. *japonicum* + 50% NPK	8.321b	6.187b	–	–	–	8.280bc	6.152c	–	–	–
	Mycorrhiza + 50% NPK	8.205c	6.152bc	80.0c	38.4c	27.82c	8.311b	6.283ab	80.0c	26.8f	26.0b
	Mixture + 50% NPK	8.201c	6.187b	79.1c	33.4d	21.90e	8.227 cd	6.278ab	78.1d	33.8c	20.5c
**Fertilization**		***	***	***	***	***	***	***	***	***	***
**Drought**		***	***	***	***	***	***	***	***	***	***
**Fertilization × Drought**		**	**ns**	***	***	***	**ns**	***	***	***	***

(−) means no result was detected, Means sharing the same letters, for a parameter during a year, do not differ significantly at *α =* 0.05 after Student–Newman–Keul test; ns, not significant; and *, **, ***, denote significant differences at *P ≤ 0.05*, *0.01*, and *0.001*, respectively, among the studied factors. Mixture (*B. japonicum* + Mycorrhiza); *CK* (Control); *D1* [irrigation withholding at early pod stage (50 days from sowing, R3)]; *D2* [irrigation withholding at seed development stage (90 days from sowing, R3)]; *F* (Frequency of root colonization); *M* (Intensity of cortical colonization) and *A* (Arbuscule frequency in roots)

**Fig. 6:**
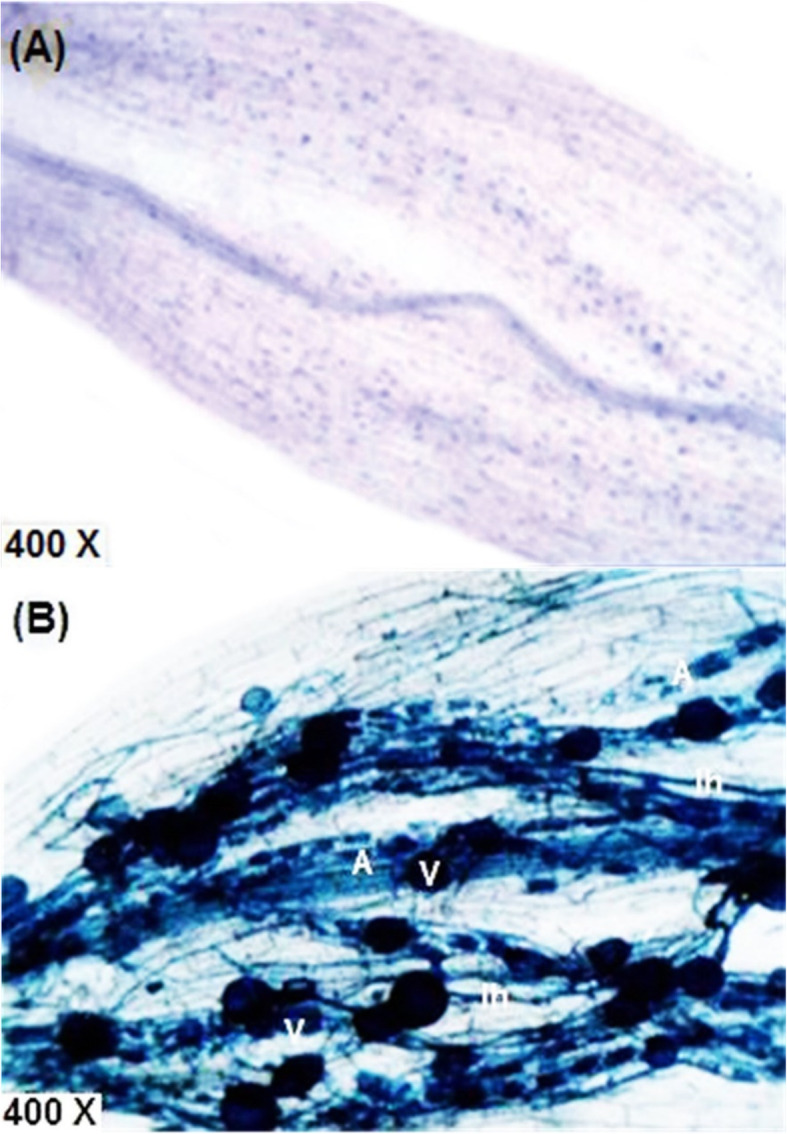
Soybean root showing mycorrhizal colonization structures. Non-treated control root (**a**), and AMF-colonized root (**b**). Ih; internal hyphae, V; vesicle, and Ar; arbuscule

The development of mycorrhiza during plant growth was monitored by using specific variables such as frequency of mycorrhizal colonization, intensity of mycorrhizal colonization and arbuscular frequency were significantly affected by the different treatments (Table 4). The root colonization of arbsucular mycorrhiza fungi was increased by the single inoculation with mycorrhiza and/or in combination with *Bradyrhizobium* (Mixture+ 50% NPK) in both growing seasons. Also, it was observed that all the estimated variables of mycorrhizal colonization were decreased in soybean roots in response to the level of the drought stress. However, mycorrhizal colonization of the plants exposed to restrained irrigation at R5 stage was higher than those exposed to withholding irrigation at R3 stage (Table 4). Different structures like arbuscules, vesicles, and internal hyphae were observed in trypan blue-stained roots of soybean plants (Fig. 6). AM fungal colonization was not observed in soybean plant roots that were not inoculated with mycorrhiza.

### Effects of biofertilizers on nuclear DNA content under drought treatments

**Fig. 7:**
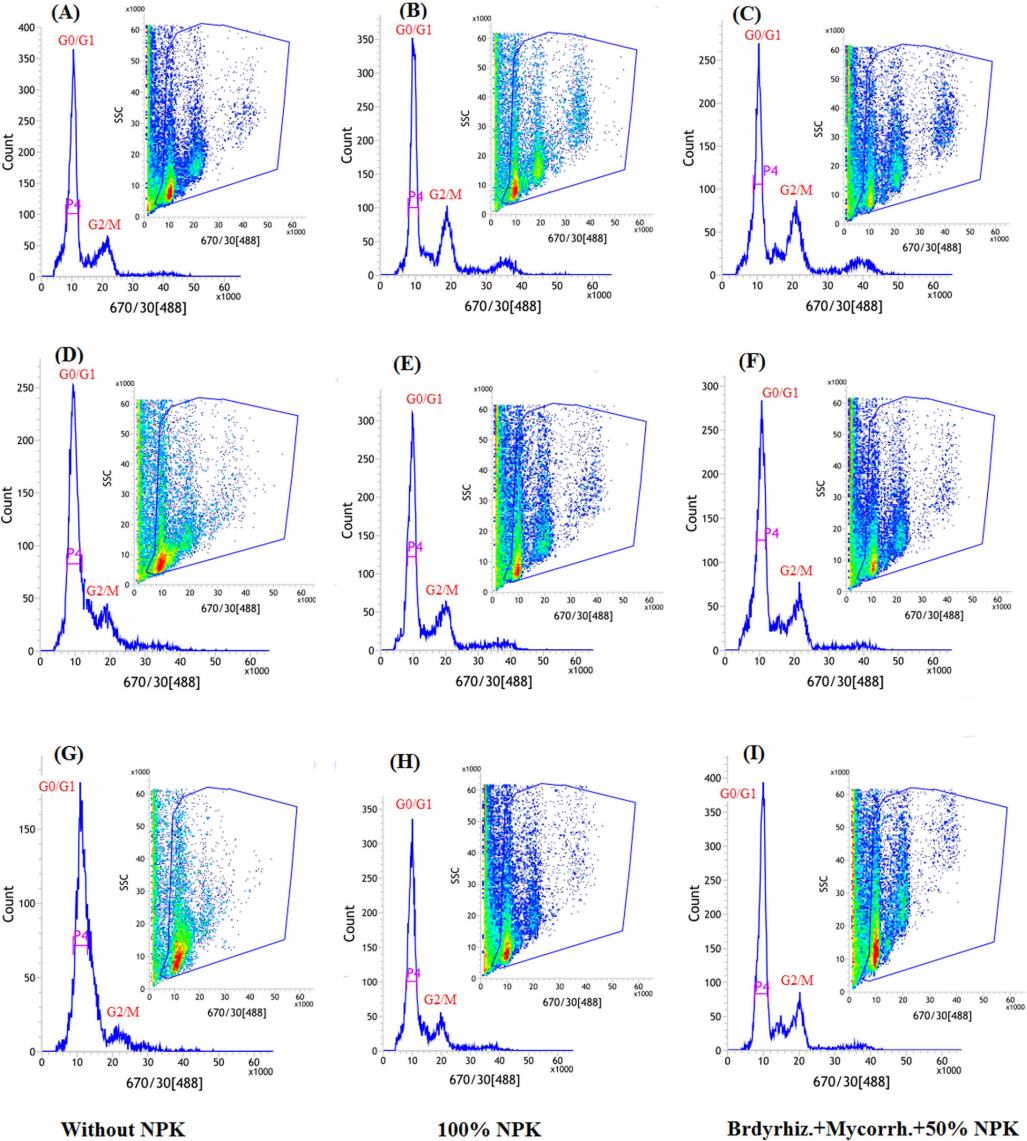
Flow cytometric analysis of soybean seed under normal irrigation (Ck) (**a**-**c**); exposed to withholding irrigation at R3 stage (**d**-**f**) and exposed to withholding irrigation at R5 stage (**g**-**i**) in 2018 growing season. Bradyrhiz. (*B. jabonicum*); Mycorrh. (Mycorrhiza)

**Fig. 8:**
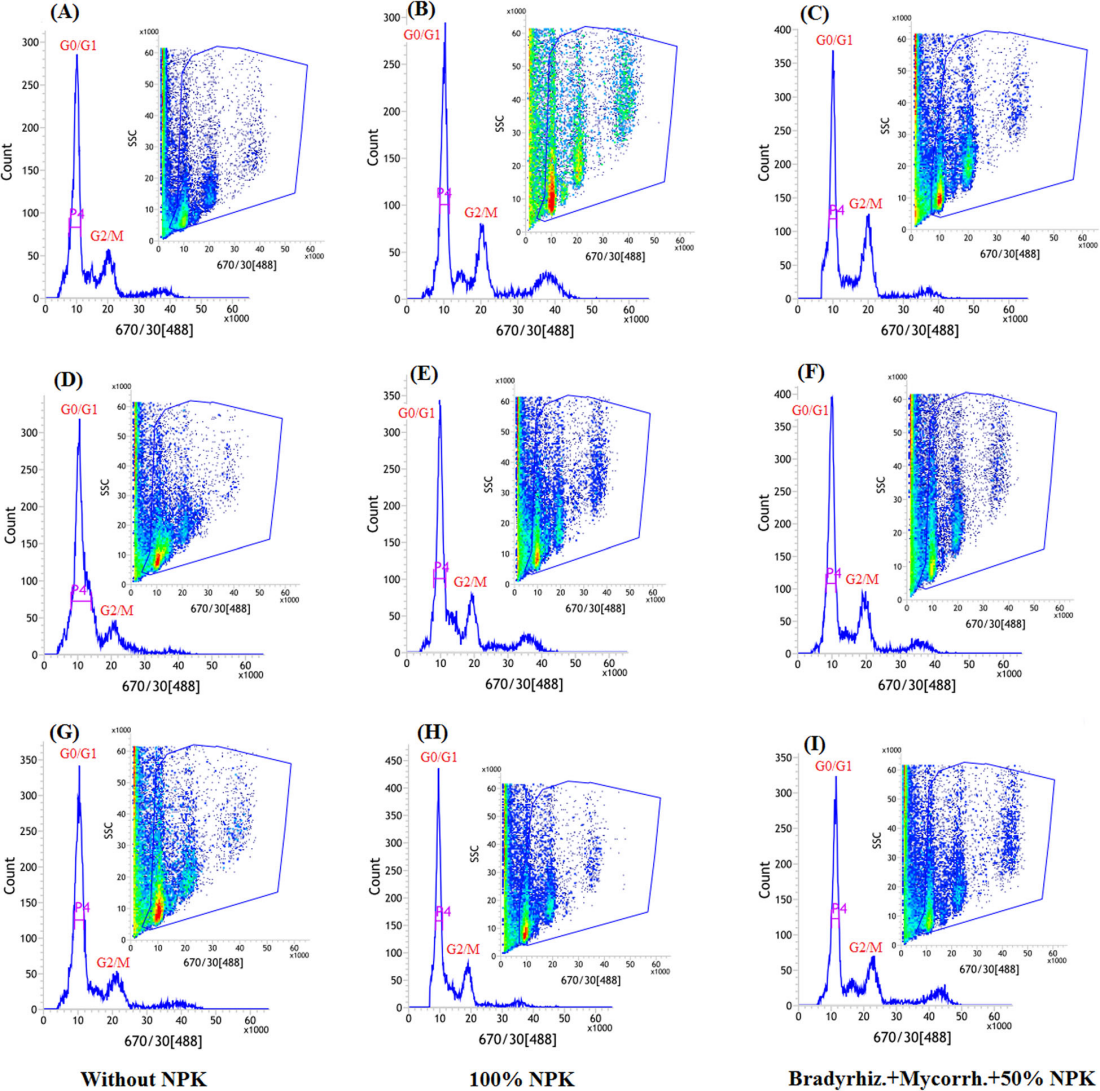
Flow cytometric analysis of soybean seed under normal irrigation (Ck) (**a**-**c**); exposed to withholding irrigation at R3 stage (**d**-**f**) and exposed to withholding irrigation at R5 stage (**g**-**i**) in 2019 growing season. Bradyrhiz. (*B. jabonicum*); Mycorrh. (Mycorrhiza)

In order to investigate whether the biofertilizers interaction could maintain the cell cycle progression of soybean under drought stress, the nuclear DNA content was analyzed using the flow cytometry technique (Figs. 7 and 8). Plants exposed to withholding irrigation at R3 and R5 stages showed changes in their cell progression as compared with unstressed-plants (Figs.7d-i and Figs. 8d-i). There were no obvious changes in the cell progression during G0/G1 stage between all treatments. On the other hand, the cells were blocked at the G2/M phase in the plants exposed to restrained irrigation at R5 stage, which was more pronounced in the plants without biofertilizers treatments (Figs. 7 and 8(g-i)).

## Discussion

Recently, biofertilizers produced from *Bradyrhizobium japonicum* and/or AMF have been demonstrated to have significant benefits for soybean growth and yield in field trials under water deficit conditions [57]. However, most studies focused mainly on the evaluation of the quantity and quality of the soybean yield. In the present study, not only the yield has been studied but also the impact of these biofertilizers on bacterial distribution and enzymatic activity in soil, different physiological, biochemical, and molecular aspects as well as the cell development of soybean plant to gain a better understanding the mechanisms of response for improving soybean adaptation and tolerance to drought stress conditions.

The ability of *B. japonicum* USDA-110 to solubilize phosphate and produce indole acetic acid was also tested in the current study. The results showed that *B. japonicum* USDA-110 reached its maximum activity after 14 days of incubation and then decreased with the advance of the incubation period (Fig. 2). The decrease in P content with the advance of the incubation period could be attributed to the utilization of P resulting in the fluctuating levels of P release, and availability of soluble P in the culture medium might also have an inhibitory effect on further P solubilization [29]. Excretory toxic products may also be responsible for such decline in P solubilization. Moreover, *B. japonicum* USDA-110 can effectively produce IAA (Fig. 2), in addition; *B. japonicum* USDA-110 was shown to make nodules on the roots of soybean plants effectively. All these characteristics of plant growth promotion may contribute to improving the growth of soybean plants.

Total bacterial counts and phosphate-solubilizing bacteria were increased in the samples of rhizosphere of the plants those treated with biofertilizers and were higher than those of chemical fertilizer-treated plants. In the present study chemical fertilizers were applied in all variants but at different dosages. The increase in bacterial count might be due to nutrient viability in the rhizosphere of biofertilizers-treated plants which supplies the required energy for soil microorganisms to decompose organic matter. Mycorrhization may also decrease the release of root exudates. Also, it was observed that drought stress in both R3 and R5 stages resulted in an increase in bacterial counts, and this might be due to soil drying and rewetting which could make a wide change in the composition of organic matter and its particles, making it more susceptible to microbial activity [58]. Additionally, the microorganisms not necessarily die under drought stress, but may enter into dormant stages to be rapidly degradable organic materials by microorganisms after rewetting. Similarly, Meisner et al. [58] and Lovieno and Baath [59], found that rewetting the dry soil stimulates bacterial growth and respiration immediately upon rewetting or exponentially after a lag period. Similar findings were also observed by Bloem et al. [60], indicating an increase in respiration, N mineralization, and bacterial growth rates after soil re-irrigation.

In this study, the inoculation of mycorrhiza alone or in combination with *Bradyrhizobium* has improved mycorrhizal colonization levels in the root tissues of soybean plants. This high colonization resulted in an increased root system in mycorrhizal treatments (Table 2) which can contribute to more nutrients and water uptake by soybean plants. However, there was a decrease in mycorrhizal colonization levels under drought but the used mycorrhizal species still have the ability to colonize soybean roots (Table 4). Similarly, Juge et al. [61] reported that combined treatment with *Bradyrhizobium* and AMF enhanced mycorrhization in the root systems. In addition, Pavithra and Yapa [62] observed a decrease in mycorrhizal colonization in soybean roots under the drought stress, also, Asrar et al. [48] found that both intensity of mycorrhizal colonization (M) and arbuscular frequency (A) were significantly decreased by 20% in the root tissue as affected by stress conditions, however, mycorrhiza still can colonize the root system. This finding suggested that the used mycorrhizal species have the ability to colonize soybean roots under drought stress and have the ability to change specific root length, root architecture and enhance plant growth by improving phosphorus nutrition and water absorption through their hyphae.

The high microbial activity in the soybean rhizosphere activated the soil enzymes mainly phosphatase and dehydrogenase in the rhizosphere of biofertilizers treated plants under both well-watered and drought stress treatments (Figs. 3a and b). The dehydrogenase activity is present in viable cells and basically dependent on the metabolic state of the soil microbial community, it can be considered a direct measure of soil microbial activity. However, soil phosphatase enzyme is essential in the mineralization of organic P [29]. The high dehydrogenase and phosphatase have a significant role in the decomposition of organic matter and the translocation of nutrients in the soil [63], and this high activity may be due to the mechanisms of bacteria and AMF in improving the physical and chemical soil properties, especially the soil structure, which enhance the microbial activity in the soil. These results are in agreement with those obtained by Gao et al. [29] and El-Sawah et al. [64] who observed an increase in the activities of phosphatase and dehydrogenase enzymes with application of biofertilizers containing PGPR and/or AFM which is associated with the bacterial counts in the rhizosphere.

In the present study, the morpho-physiology and yield of soybean has negatively affected by withholding water at R3 and R5 stages, however, soybean growth was improved by biofertilizers treatments in both growing seasons. This might be due to the ability of *Bradyrhizobium* to fix nitrogen, solubilize phosphate and produce IAA (Fig. 2), which can be coupled to the improvement in plant growth, moreover AM symbiosis increased the rate of plant growth by increasing the concentration of nutrients in particular P in plant tissues. In addition, the increase in biological nitrogen fixation and the relatively decreased uptake of nitrate from the soil, are acidifying the rhizosphere more intensively, which might contribute to the mobilization of phosphates from the soil. Jayne and Quigley [65] reported that mycorrhiza improved plant growth under water-deficit condition, which might be due to the capability of AMF to improve phosphorus nutrition content, water acquisition as well as cellular signaling of the plant under drought stress [66]. In the current study, a significant reduction in grain yield was observed in the plants those exposed to withholding irrigation at R3 stage as compared with withholding irrigation at R5 stage and control in both growing seasons. However, inoculation with *Bradyrhizobium*, AMF, and their combination resulted in an increase in grain yield under drought stress. Previously, Soe et al. [67] reported that combined use of *B. japonicum* had a significant effect on the grain yield of soybean as compared with the control plants. These results are consistent with recent findings, reporting that the use of a biofertilizer produced from effective *Bradyrhizobium* significantly increased grain yield in both soybean and mung bean [43].

The current results showed that inoculation with *B. japonicum* alone or in combination with AMF has a significant increase in soybean nodulation (Table 3). Several studies have reported the positive effect of *B. japonicum* and AMF on soybean nodulation. In this regard, Hao et al. [68] reported that AM fungus inoculation improved mycorrhiza plant–rhizobium symbiosis and this was more effective for promoting plant growth during drought stress. Moreover, AMF could also decrease oxidative stress occurring in the nodules [69], and improve the carbon metabolism of nodules [70], which may increase the effectiveness of the nodule to fix atmospheric nitrogen. Soe and Yamakawa [71], found that the co-inoculation with *B. yuanmingense* P4 significantly improved the nodules’ number of soybean. Our results showed that *B. japonicum* alone recorded higher nodules number as compared with AMF alone or the mixture treatments. Some studies also reported similar findings that the individual inoculation with biofertilizers proved to be more effective than the combined inoculation, this may strongly depend on the type of microorganisms that are combined. As such, Juge et al. [61] found that triple treatment **(***Bradyrhizobium × Azospirillium ×* AMF) and the dual treatment (*Bradyrhizobium ×* AMF) had fewer nodules than *Bradyrhizobium* alone*.* Interestingly [72] reported that the combined use of bradyrhizobial strains and *S. griseoflavus* P4 increased nodulation as compared to the individual treatment. Also, Htwe et al. [43] reported that a biofertilizer mixture containing *B. japonicum*, *B. elkanii* and *S. griseoflavus* enhanced nodules number and nodules dry weights of soybean. The present study suggested that the inhibition of the nodulation was higher in the plants those exposed to withholding irrigation at R3 stage, which may be due to the inhibition of the carbon assimilation and nitrogen metabolism under drought stress. Previously, Miao et al. [73] reported similar results that nodules number per plant was decreased by water stress at the flowering stage than that at the pod bearing and grain filling stages.

In the present study, both drought stress and biofertilizers treatments showed an additive up-regulation of *CAT* and *POD* activities and their related genes expression which indicated the post transcriptional activation of corresponding enzymes activities that could scavenge different ROS in plant cells under the stress condition. A similar result was also observed by Mittler [74] who stated that an increased level of antioxidants has a pivotal role in deteriorating the ROS activity, thus plants could be able to maintain their physiological functions under the stress environment. Previously, Salah et al. [53] reported that POD enzyme can serve as an intrinsic defense tool to resist oxidative stress in rice plants and also can be used as a potential biomarker to evaluate the intensity of stress [75]. Interestingly, a previous study reported that the activity of CAT was related to the content of soil organic matter and the number of soil microbes [76]. Accordingly, it seems that inoculation of soybean plants with biofertilizers activated the antioxidant enzymes which contribute to the scavenge of the oxidative stress induced by the lipid peroxidation under drought stress.

The proline content in the present study was increased in the plants under drought stress (Fig. 3f). Several studies have observed accumulation of proline under different abiotic stresses. As such, Kim and Tai [77] found a significant increase in the proline content under the chilling stress, stating that proline has the function to increase adaptation of rice under cold stress. The present study reports that the proline metabolism-related genes were up-regulated under the drought stress (Figs. 5c-f), which was consistent with the proline content (Fig. 3f). Our findings are consistent with a previous study reporting that proline content was improved under osmotic stress due to the up-regulation of the gene encoding *P5CS* [78]. Moreover, Dobra et al. [79] observed up-regulation of *P5CS* gene expression in the leaves of tobacco plants exposed to drought stress for 6-days. Interestingly, Hien et al. [80] indicated that P5CS activity is not responsible for the differential proline accumulation in plants that have different levels of abiotic stress tolerance. Several other studies also indicated that the proline accumulation and the transcript level of P5CR was not affected under different abiotic stresses [81, 82]. There is some evidence that salinity stress results in up-regulation of *P5CR* in different plant species such as soybean, wheat, *Arabidopsis*, and pea [83]. The current study suggested that the genes involved in proline metabolism are the main key to control the level of proline and maintain the lower level of the proline degradation under drought stress. This evidence is in accordance with a previous study in which it has been reported that the *P5CDH* and *PDH* genes are the primary regulators of the proline oxidation that is required to maintain the cellular ROS balance [84], and also are necessary to control the proline levels under abiotic stress [82].

The current finding revealed that changes in the cell progression of soybean roots at both R3 and R5 stages were observed under different biofertilizers treatments (Figs. 7 and 8). These findings suggested that biofertilizers treatments diminished the inhibition effects of drought on cell progress and resulted in a shorter time for cell accumulation and cycle division. The reduction of the cell production in the drought-stressed plants might be due to a smaller number of dividing cells such as a meristem size reduction [85, 86]. Also Sheteiwy et al. [13] reported that the nuclear accumulation, especially at G0/G1 stage, was inhibited under salinity and osmotic stress and their combination. A previous study suggested that cell cycle activities are involved in the stress response mediated by transcription factors [87]. The current study explored that the changes in the cell cycle in the biofertilizers-treated plants might be due to feedback of the cell to the stress condition and to increase plant adaptation to drought stress. The feedback of the cell cycle might be controlled by the positive and negative regulation of the expression of some cell cycle genes which leads to perturbation of cell cycle progression in response to abiotic stresses [85].

## Conclusions

**Fig. 9:**
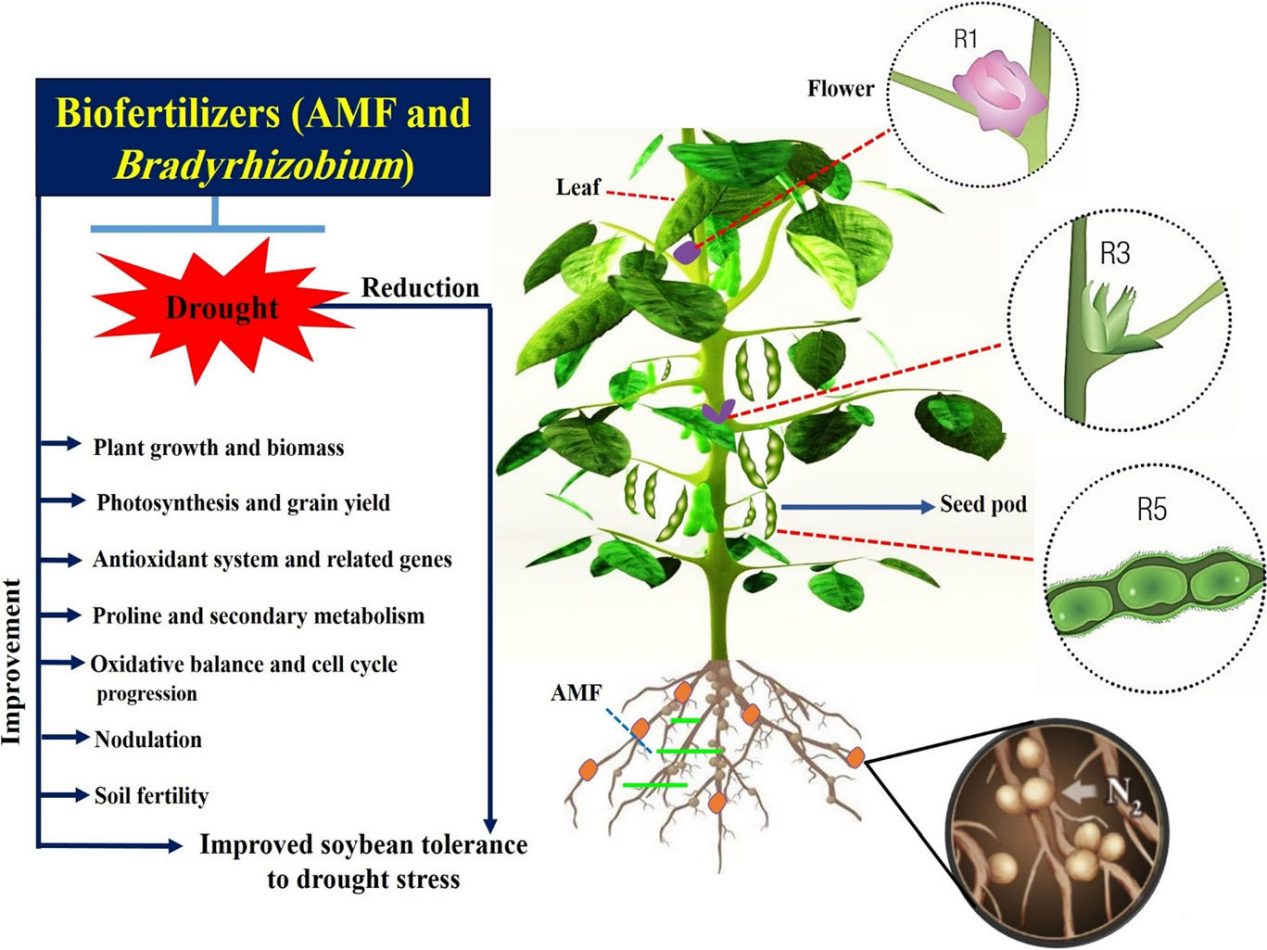
A schematic mechanism showing the interaction between soil, plant and microorganisms to improve the growth and yield of soybean under drought stress condition

In conclusion, a significant increase in plant biomass, chlorophyll content, nodulation and grain yield of well-watered soybean plants as compared with plants exposed to drought stressed at R3 and R5 stages. Inoculation with AMF and *Bradyrhizobium* improved the growth and yield of soybean under drought stress conditions. Bacterial counts, mycorrhizal colonization levels, and activities of soil enzymes were also increased in rhizosphere soil of plants those treated with biofertilizers which may have a significant role for improving growth and yield of soybean under water deficit condition. Biofertilizers have improved the antioxidant system of soybean and their related genes which contribute to reducing the oxidative damage induced by drought stress. In addition, the accumulation of proline and up-regulation of their related metabolism genes in the plants might play a vital role as a stress signal influencing adaptive responses of soybean under drought stress. In addition, the plants that were inoculated with biofertilizers have accumulated higher activities of the secondary metabolism which was consistent with their expression pattern under drought stress. Biofertilizers treatments diminished the inhibition of drought stress effects on cell progress and resulted in a shorter time for DNA accumulation and cycle division. This study concluded that application of biofertilizers in association with soybean plants can be used as a promising and alternative technology to alleviate water stress effects on soybean growth performance, which may help to obtain greater sustainability of the agro ecosystems by introducing it into agricultural systems to improve soil fertility, plant growth under drought stress. This approach gives a detailed view (Fig. 9) of the effect of an inoculant on the soil ecosystem’s functioning.

## Supplementary Information

**Additional file 1: Table S1.** Sequences of oligonucleotide primers used in QRT-PCR.

## Data Availability

All data generated or analyzed during this study are included in this published article.
