# Supplementary material for: Physiological and biochemical responses of soybean plants inoculated with Arbuscular mycorrhizal fungi and Bradyrhizobium under drought stress
Source: BMC Plant Biol. 2021 Apr 22;21:195. doi: 10.1186/s12870-021-02949-z (PMC8061216; doi:10.1186/s12870-021-02949-z)
Supplement: Supplementary file 1 — Additional file 1: Table S1. Sequences of oligonucleotide primers used in QRT-PCR. [file 12870_2021_2949_MOESM1_ESM.docx]

Physiological and biochemical responses of soybean plants inoculated with Arbuscular mycorrhizal fungi and *Bradyrhizobium* under drought stress

Mohamed S. Sheteiwy^1,2*^, Dina Fathi Ismail Ali^3^, You-Cai Xiong^4^*, Marian Brestic^5,11^, Milan Skalicky^5^, Yousef Alhaj Hamoud^6^, Zaid Ulhassan^7^, Hiba Shaghaleh^8^, Hamada AbdElgawad^9^, Muhammad Farooq^10^, Anket Sharma^12^ and Ahmed M. El-Sawah^3*^

^1^Salt-Soil Agricultural Center, Institute of Agriculture Resources and Environment, Jiangsu Academy of Agricultural Sciences (JAAS), Nanjing 210014, China

^2^Department of Agronomy, Faculty of Agriculture, Mansoura University, Mansoura 35516, Egypt

^3^Department of Agricultural Microbiology, Faculty of Agriculture, Mansoura University, Mansoura 35516, Egypt

^4^State Key Laboratory of Grassland Agro-ecosystems, Institute of Arid Agroecology, School of Life Sciences, Lanzhou University, Lanzhou 730000, China

^5^Department of Botany and Plant Physiology, Faculty of Agrobiology, Food and Natural Resources, Czech University of Life Sciences Prague, Kamycka 129, 16500 Prague, Czech Republic

^6^College of Agricultural Science and Engineering, Hohai University, Nanjing, China

^7^Institute of Crop Science and Zhejiang Key Laboratory of Crop Germplasm, Zhejiang University,

Hangzhou 310058, China

^8^College of Chemical Engineering, Nanjing Forestry University, Nanjing 210037, China

^9^Department of Botany, Faculty of Science, University of Beni-Suef, Beni-Suef 62511, Egypt

^10^Department of Plant Sciences, College of Agricultural and Marine Sciences, Sultan Qaboos University, Al-Khoud 123, Oman

^11^Department of Plant Physiology, Slovak University of Agriculture, Nitra 94911, Slovakia

^12^State Key Laboratory of Silviculture, Zhejiang A&F University, Hangzhou, China

*Correspondence to: MS Sheteiwy (*Department of Agronomy, Faculty of Agriculture, Mansoura University, Mansoura 35516, Egypt*); AM El-Sawah (*Department of Agricultural Microbiology, Faculty of Agriculture, Mansoura University, Mansoura 35516, Egypt*) and YC Xiong (*Institute of Arid Agroecology, School of Life Sciences, Lanzhou University, Lanzhou 730000, China*). E-mail: [salahco_2010@mans.edu.eg](mailto:salahco_2010@mans.edu.eg) (MS Sheteiwy); [ahmedelsawah89@mans.edu.eg](mailto:ahmedelsawah89@mans.edu.eg) (AM El-Sawah); [xiongyc@lzu.edu.cn](mailto:xiongyc@lzu.edu.cn) (YC Xiong)

**Table S1.** Sequences of oligonucleotide primers used in QRT-PCR.

| Gene | Primer orientation | Sequence (5ʹ-3ʹ) |
| --- | --- | --- |
| *CAT* | Forward | AGCATCTCACCTGAACTTGAA |
|  | Reverse | AGGTGAGAGGTTTGTGGCC |
| *POD* | Forward | TTGAAATAAACCAAAGGAGTAGT |
|  | Reverse | AATAATTATTTGAATCTCTTTAAGG |
| *GmP5CS* | Forward | ATTCCTGTCCTGGGTCATGCAGAT |
|  | Reverse | AAGAGTTTCCATGGCATTGCAGCC |
| *GmP5CR* | Forward | TGACAGTAATCCCACCCAGCTCAA |
|  | Reverse | ACTTCAGAACCAGGTTGGGTCCAT |
| *GmPDH* | Forward | TCAACTTGCCAACCAGAGACTCCT |
|  | Reverse | ATCGATAGCCGGTTAACTGTGGT |
| *GmP5CDH* | Forward | TAGGGCGACTATGGTAATTGCGGT |
|  | Reverse | TGCCCACAGTGTCGAAACGGAATA |
| GmSPS1 | Forward | AAGCAGCTGGAGAGTGAGACAG |
|  | Reverse | TCCGACAAGTCCTCAGACATATCC |
| GmSuSy1 | Forward | AGAAGTGCAAGCTTGACCCAACTC |
|  | Reverse | ATACGCTGGAGACCAGCCTTTG |
| GmC-INV | Forward | CGTTGCCATCCTCCTTCAGATAG |
|  | Reverse | AGGGATAGAGCAGTGAGAGCTG |
| Actin gene | Forward | AAGTGCTTCTAAATTGTTTGGTT |
|  | Reverse | TGACAATGACATTGCAGAGAAT |
